# Trigeminal Herpes Zoster With Mucosal Involvement in a Patient on Prophylactic Valacyclovir: An Uncommon Presentation

**DOI:** 10.7759/cureus.68859

**Published:** 2024-09-07

**Authors:** Bret-Ashleigh Coleman, Kelly M Frasier, Mary Grace Hash, George B Tisdale

**Affiliations:** 1 Dermatology, Edward Via College of Osteopathic Medicine, Auburn, USA; 2 Dermatology, Northwell Health, New Hyde Park, USA; 3 Dermatology, University of Alabama at Birmingham School of Medicine, Birmingham, USA

**Keywords:** atypical presentation, herpetic neuralgia, mucosal involvement, prophylactic valacyclovir, trigeminal herpes zoster

## Abstract

This case report details a unique presentation of trigeminal herpes zoster with mucosal involvement in a 72-year-old female undergoing prophylactic valacyclovir treatment for suspected herpes labialis. The patient initially presented for a routine skin examination. Despite the absence of clinical evidence of herpes labialis, she was prescribed prophylactic valacyclovir. Three days later, the patient developed a unilateral rash, gingival darkening, and severe ipsilateral tooth pain. An examination revealed a crusted erythematous rash along the mandibular division of the left trigeminal nerve and a darkening of the gums. Diagnosed with trigeminal herpes zoster, she was treated with valacyclovir and a prednisone taper. A follow-up showed the resolution of the skin and gingival lesions, though herpetic neuralgia persisted. This case underscores the complexity and potential atypical presentations of herpes zoster, even under prophylactic antiviral therapy. It highlights the importance of considering herpes zoster in differential diagnoses, especially in patients presenting with unilateral dermatological and mucosal symptoms.

## Introduction

Herpes zoster, commonly known as shingles, is caused by the varicella-zoster virus (VZV) reactivation, which remains dormant in the dorsal root ganglia following a primary varicella (chickenpox) infection. The incidence of herpes zoster increases with age and immunosuppression, with approximately one-third of the population experiencing an episode in their lifetime [1]. The risk significantly escalates in individuals over 60 years due to the decline in cell-mediated immunity [2]. Characterized by a painful, unilateral vesicular rash typically confined to a single dermatome, herpes zoster can lead to various complications, including postherpetic neuralgia (PHN), a chronic pain condition that persists long after the rash has healed.

In the United States, approximately one million cases of herpes zoster are reported annually, with an incidence rate of three to five per 1,000 person-years among the general population. This rate increases significantly to six to 12 per 1,000 person-years in individuals aged 60 and older, a trend attributed to the natural decline in cell-mediated immunity within this age group [3]. Beyond the elderly, immunocompromised individuals, such as those undergoing chemotherapy, organ transplant recipients, and patients with HIV, are particularly vulnerable to the disease. The introduction of the shingles vaccine has been a pivotal development in mitigating the incidence and severity of herpes zoster and its associated complications in older adults [4]. Nevertheless, it is crucial to recognize that one does not need to be immunocompromised or have comorbidities for the reactivation of the virus to occur. 

Trigeminal herpes zoster is a specific form of the disease when VZV reactivates in the trigeminal ganglion, affecting the ophthalmic, maxillary, or mandibular branches of the trigeminal nerve. This manifestation can lead to severe pain, vesicular eruptions, and if untreated, complications such as vision loss when the ophthalmic division is involved [5]. Although less common, mucosal involvement presents additional diagnostic challenges and may complicate the clinical picture further. Oral lesions, as seen in this case, indicate the spread of the virus along the branches of the trigeminal nerve, causing significant discomfort and potential for secondary infections.

The patient described in this case report presented with a unique clinical scenario: despite being on prophylactic valacyclovir, typically effective in preventing herpes simplex virus (HSV) outbreaks, she developed a severe form of herpes zoster involving the trigeminal nerve and oral mucosa. This raises questions about the efficacy of antiviral prophylaxis in preventing herpes zoster reactivation, particularly in elderly patients with multiple comorbidities. This case underscores the need for heightened clinical vigilance and potentially a reevaluation of prophylactic strategies in such populations.

## Case presentation

A 72-year-old female patient presented to the office for an annual full-body skin examination with a chief complaint of an oncoming episode of herpes labialis. She described a tingling and burning sensation on her left oral commissure similar to previous episodes of herpes labialis that began 24 hours prior. She denied any symptoms such as arthralgias or headache. Her past medical history was notable for hypertension, well controlled with 20 mg of rosuvastatin once daily, and herpes labialis. She was previously given valacyclovir for prophylaxis but did not have an active prescription. Upon physical examination, there was no evidence of herpes labialis. Due to the similarity of previous episodes, a prophylactic dose of 2000 mg of valacyclovir twice daily was given. 

**Figure 1:**
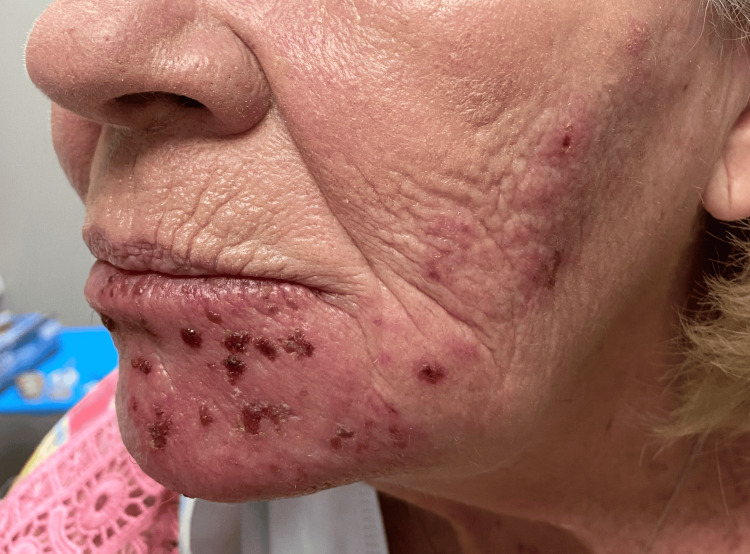
Crusted vesicular rash on an erythematous base along the mandibular division of the left trigeminal nerve.

**Figure 2:**
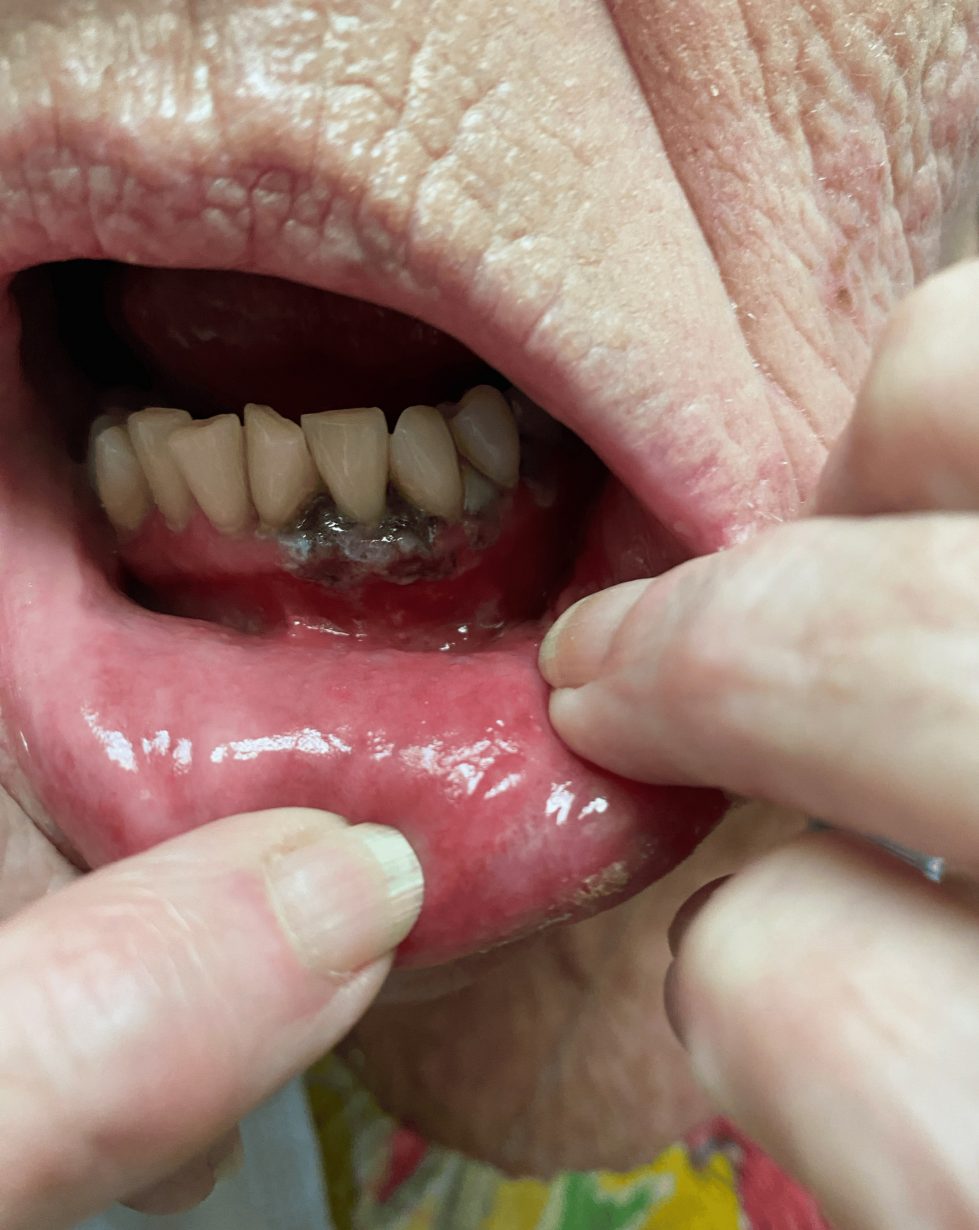
Darkening of ipsilateral gums, consistent with mucosal involvement.

Three days after the initial visit, the patient returned to the office for a new onset facial rash that had been present for 48 hours. Physical exam revealed crusted vesicles on an erythematous base in the mandibular nerve distribution, as seen in Figure 1. Upon further examination, congruent unilateral gum darkening was observed, as seen in Figure 2. She endorsed unilateral facial pain, rated 7/10 on a pain scale, as well as odontalgia that extended from the left central incisor to the left molars. Poor periodontal health was not considered a factor for the odontalgia and associated neuralgia, as the odontalgia was absent both before and after the resolution. Moreover, the pain exhibited the characteristic shooting and burning quality of the zoster. No further lesions were seen throughout the oral mucosa. Given the patient's presentation of a new onset facial rash, unilateral facial pain, and odontalgia, the differential diagnosis initially included acute herpetic gingivostomatitis, trigeminal neuralgia, dental abscess, and bacterial cellulitis. However, the presence of crusted vesicles in a dermatomal distribution, along with the characteristic shooting and burning pain, strongly pointed to trigeminal herpes zoster, which was ultimately confirmed with mucosal involvement. She was given 1 gram of valacyclovir three times a day for seven days and a 60 mg prednisone taper for pain and inflammation. Due to the oral involvement, she was advised to see her dentist to rule out ischemic necrosis of the gums and return for follow-up in seven days.

**Figure 3:**
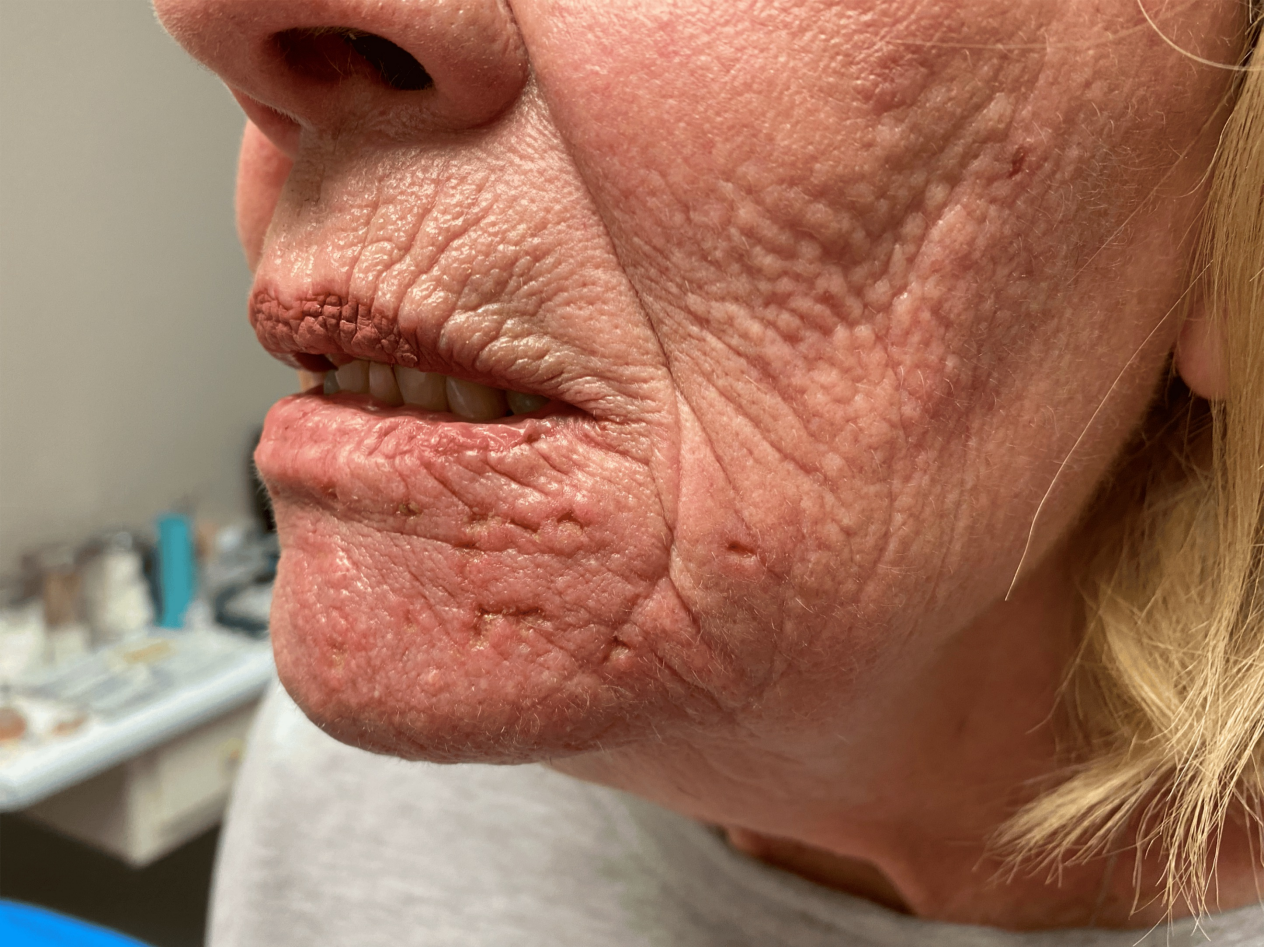
Moderate resolution of mandibular distribution of herpes zoster with mild pitting and post-inflammatory hyperpigmentation.

**Figure 4:**
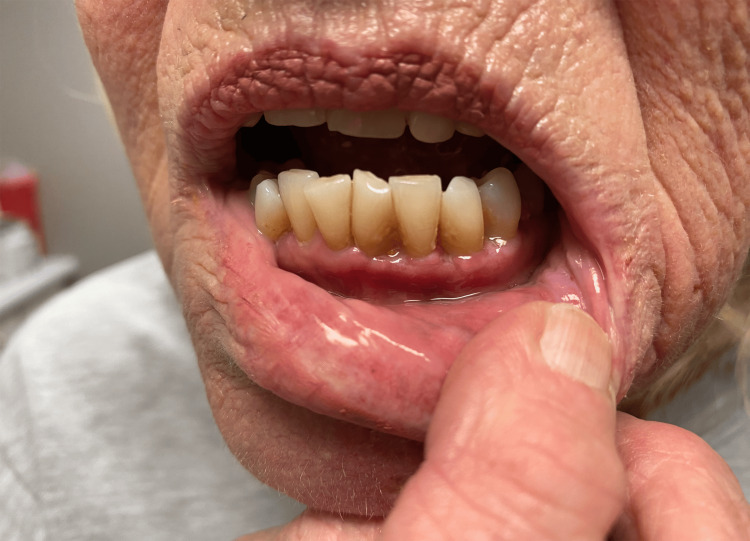
Complete resolution of oral mucosal involvement.

Ten days after the initial visit, the patient returned for follow-up care. A complete resolution was seen of the oral mucosa (as indicated in Figure 3) with a significant reduction in odontalgia. Due to the rapid resolution of symptoms, the patient was not examined by a dental practitioner. A skin examination revealed mild erythema with marked improvement of the vesicular rash, as seen in Figure 4. Postherpetic neuralgia was present in the mandibular nerve distribution. Two months after the initial visit, the patient’s post-herpetic neuralgia was completely resolved, and her skin and oral mucosa were at baseline. 

## Discussion

This case report presents a rare and intriguing manifestation of herpes zoster, characterized by darkening of the gums and severe unilateral tooth pain. The bluish-black discoloration of the gums observed in this patient is an uncommon finding not typically associated with herpes zoster, adding a unique dimension to our understanding of the disease’s oral manifestations. This unusual presentation warrants a closer examination of the potential mechanisms and clinical implications of mucosal involvement in herpes zoster.

The pathophysiology underlying the bluish-black discoloration of the gums likely involves VZV-induced vasculitis, which can cause localized ischemia and subsequent necrosis of the gingival tissues. The involvement of the trigeminal nerve, particularly the mandibular division, facilitates extensive viral spread to the oral mucosa, resulting in significant tissue damage and pigmentation changes. This case suggests that the virus’s impact on the vascular supply to the gingiva may lead to hemorrhagic necrosis, presenting as darkened gums [6,7]. This finding is particularly notable because it expands the spectrum of oral manifestations of herpes zoster, which typically includes ulcerations, vesicular eruptions, and rarely osteonecrosis [8]. While osteonecrosis of the gingiva with subsequent tooth exfoliation is rare but well-documented in the literature, our patient's suspected necrosis was self-resolving and did not lead to exfoliation. Additionally, no other mucosal involvement was observed in this patient.

Tissue necrosis typically requires debridement, making the spontaneous resolution of the bluish-black discoloration of the gums observed in this patient both unexpected and clinically significant. While the initial viral insult can cause severe and alarming mucosal changes, the body’s regenerative capacity can reverse these changes with appropriate antiviral and supportive therapy. This outcome suggests that the necrosis, in this case, was limited, and the oral mucosa could heal without debridement, which is unusual as necrotic areas of the skin do not typically return to healthy tissue without intervention. The areas where tissue death began to occur were able to regenerate and recover with supportive therapy, and they did not worsen, highlighting the effectiveness of antiviral therapy in managing the mucosal manifestations of herpes zoster. This case aligns with the classification of necrotizing periodontal disease, where mild or superficial necrosis, especially in immunocompromised patients, can be treated with supportive measures and treatment of the underlying disease [9]. The resolution of gum discoloration without surgical intervention or extensive dental procedures underscores the efficacy of antiviral therapy in managing the mucosal manifestations of herpes zoster. This emphasizes the importance of timely medical intervention and highlights the potential for recovery, even in cases with severe mucosal involvement. Similar findings were reported by Matsuda et al., where early antiviral treatment and supportive oral health management facilitated recovery in a patient with extensive oral ulceration due to herpes zoster [10]. Their case demonstrated the critical role of multidisciplinary care, involving both medical and dental professionals, in managing severe oral manifestations of herpes zoster. 

This unique case also underscores the need for heightened awareness among clinicians regarding the diverse presentations of herpes zoster. Dental professionals, in particular, should be vigilant in recognizing such atypical manifestations, as early diagnosis and treatment can significantly impact patient outcomes. Integrating dental care into the management plan for patients with herpes zoster involving the trigeminal nerve can provide comprehensive care, addressing the disease’s immediate and long-term complications. 

Furthermore, this case highlights the importance of considering herpes zoster in the differential diagnosis of unexplained oral pigmentation, particularly in elderly patients. As the aging population grows, the incidence of herpes zoster is likely to increase, making it essential for clinicians across disciplines to be familiar with its varied presentations [3]. This awareness can facilitate prompt diagnosis and treatment, potentially preventing severe complications such as postherpetic neuralgia.

## Conclusions

In summary, the bluish-black discoloration of the gums in this patient with herpes zoster represents a novel and rare manifestation of the disease. The spontaneous resolution of this pigmentation underscores the potential for recovery with appropriate treatment and highlights the importance of a multidisciplinary approach in managing such cases. This finding expands our understanding of the oral manifestations of herpes zoster and emphasizes the need for further research into the mechanisms and management of mucosal involvement in VZV infections.
